# Therapeutic drug monitoring-based dose optimisation of piperacillin/tazobactam to improve outcome in patients with sepsis (TARGET): a prospective, multi-centre, randomised controlled trial

**DOI:** 10.1186/s13063-019-3437-x

**Published:** 2019-06-06

**Authors:** Stefan Hagel, Sandra Fiedler, Andreas Hohn, Alexander Brinkmann, Otto R. Frey, Heike Hoyer, Peter Schlattmann, Michael Kiehntopf, Jason A. Roberts, Mathias W. Pletz, on behalf of the TARGET Study Group, Frank Bloos, Stefan Angermair, Hendrik Bracht, Eberhard Barth, Stefan Kluge, Axel Nierhaus, Johann Motsch, Thorsten Brenner, Thorsten Annecke, Friedhelm Bach, Markus Weigand, Markus Schappacher, Andreas von Ameln-Mayerhofer, Guido Michels, Anka Roehr, Thomas Fuchs, Sarah Eichner, Christina Köng, Nadine Pinder, Carsten Müller, Miriam Petersen, Peter Schlattmann, Gerald Steinbach, Dominik Jarczak, Dirk Weisman, Max Kurlbaum, Anke Braune

**Affiliations:** 1 Institute for Infectious Diseases and Infection Control, Jena University Hospital, Jena, Germany; 2 Center for Sepsis Control and Care (CSCC), Jena University Hospital, Jena, Germany; 3 Center for Clinical Studies, Jena University Hospital, Jena, Germany; 4 Department of Anaesthesiology and Intensive Care Medicine, University Hospital of Cologne, Cologne, Germany; 5 Department of Anaesthesiology and Intensive Care Medicine, General Hospital of Heidenheim, Heidenheim, Germany; 6 Department of Pharmacy, General Hospital of Heidenheim, Heidenheim, Germany; 7 Institute of Medical Statistics, Computer Sciences and Data Sciences, Jena University Hospital, Jena, Germany; 8 Department of Clinical Chemistry and Laboratory Medicine, Jena University Hospital, Jena, Germany; 9 Integrated Biobank Jena (IBBJ), Jena University Hospital, Jena, Germany; 10 University of Queensland Centre for Clinical Research, and School of Pharmacy, University of Queensland, Brisbane, Australia; 11 Department of Intensive Care Medicine and Pharmacy Department, Royal Brisbane and Women’s Hospital, Brisbane, Australia

**Keywords:** Sepsis, Therapeutic drug monitoring (TDM), Piperacillin, Pharmacokinetics, Pharmacodynamics, Continuous infusion

## Abstract

**Background:**

Sepsis is a life-threatening organ dysfunction caused by a dysregulated host response to infection with a hospital mortality in excess of 40%. Along with insufficient and delayed empirical antimicrobial therapy, inappropriate antimicrobial exposure has been identified to negatively affect patient outcomes. Receipt of prolonged infusion (i.e. extended or continuous infusion) of piperacillin/tazobactam (TZP) improves antimicrobial exposure and is associated with reduced mortality in patients with sepsis. Using therapeutic drug monitoring (TDM) with dosing tailored to the altered pharmacokinetics of the individual patient to avoid under- and overdosing may be a further strategy to improve patient outcomes. This current trial will address the question whether a TDM-guided therapy with TZP administered by continuous infusion will result in a greater resolution of organ dysfunction and hence better clinical outcome compared to continuous infusion of the total daily dose of TZP without TDM.

**Methods:**

The study is an investigator-initiated, multi-centre, parallel-group, single-blinded, randomised controlled trial. The trial will be conducted in several centres across Germany. Adult patients (aged ≥ 18 years) with severe sepsis or septic shock will be eligible for study participation. Participants will be randomly assigned to receive either TZP by continuous infusion guided by daily TDM of piperacillin (experimental group) or by continuous infusion without TDM guidance (total daily dose in normal renal function 13.5 g TZP) (control group). The pharmacokinetic (PK)/pharmacodynamic (PD) target will be 100% f T_>4MIC_ (percentage of time during a dosing interval that the free [f] drug concentration exceeds 4 times the minimum inhibitory concentration). The primary efficacy endpoint is the change in mean total Sequential Organ Failure Assessment score from day 1 after randomisation until day 10 or discharge from the intensive care unit or death, whichever comes first. Secondary outcomes include mortality, clinical cure, microbiological cure, overall antibiotic use, individual components of the primary outcome, adverse events and analysis of PK and (PD) indices.

**Discussion:**

This trial will assess for the first time whether continuous infusion of TZP guided by daily TDM in patients with sepsis will result in a greater resolution of organ dysfunction and hence better clinical outcome compared to continuous infusion without TDM.

**Trial registration:**

German Clinical Trials Register (GermanCTR), DRKS00011159. Registered on 10 October 2016.

**Electronic supplementary material:**

The online version of this article (10.1186/s13063-019-3437-x) contains supplementary material, which is available to authorized users.

## Background

Sepsis is a life-threatening organ dysfunction caused by a dysregulated host response to infection [1]. Sepsis is a leading cause of death, morbidity and expense [2]. Initial sepsis management comprises early recognition, haemodynamic resuscitation, source control and antimicrobial therapy. Empirical antimicrobial therapy not covering the causative organism and delay in administration is associated with a substantial increase in morbidity and mortality [3]. In addition, there is a growing body of evidence that an inappropriate antimicrobial exposure negatively affects clinical outcomes as well [4].

Dosing of antimicrobial agents in critically ill patients most often is based on manufacturers’ recommendations, which generally provide dosing information derived from dose-finding studies in healthy individuals or moderately ill patients. These results are then extrapolated to critically ill patients, which might be not accurate for this population [4]. Indeed, there are numerous studies showing that critically ill patients commonly develop extreme pathophysiological changes that can alter antibiotic pharmacokinetics and consequently affect drug exposure in this population. Such changes include increased volume of distribution and changes in renal and hepatic function and protein binding. In addition, the use of extracorporeal circuits makes predictions regarding total drug clearance and appropriate dosing very difficult [5]. As a consequence, numerous studies have demonstrated that antibiotic plasma concentrations are variable and unpredictable in critically ill patients, and a significant number of patients do not reach the pharmacokinetic/pharmacodynamic (PK/PD) targets, which increases the likelihood of therapeutic failures and the emergence of bacterial resistance [6–10]. In the Defining Antibiotic levels in Intensive Care Unit Patients (DALI) study [11] for example, a significant variability in beta-lactam PK/PD exposures in critically ill patients was reported, varying by more than 1000-fold. Of the 248 patients treated for infection, 16% failed to achieve even the most conservative PK/PD target of free drug concentration sufficiently exceeding the minimum inhibitory concentration (50% f T_>MIC_) with standard beta-lactam dosing, and these patients were 32% less likely to have a positive clinical outcome. Other studies suggest that up to 50% of critically ill patients are underdosed with fixed-dose antibiotic regimens [12–14]. Aggravating the problem, infections in critically ill patients are often caused by pathogens with higher minimum inhibitory concentrations (MICs), therefore requiring higher drug exposure for successful treatment of the infection [15]. Furthermore, besides the risk of underdosing, the risk of antibiotic-induced toxicity in overdosing is increasingly apparent as well. Beta-lactam antibiotics-induced toxicity may manifest in the form of neurological deterioration, renal complications or hepatic injury [16–18]. In view of such challenges for appropriate antibiotic dosing in critically ill patients, there is a strong rationale to move to an individualised dosing approach [19].

Compared to intermittent bolus dosing, as recommended in the manufacturers’ Summary of Product Characteristics, administration of beta-lactam antibiotics by prolonged infusion (i.e. extended infusion or continuous infusion) in critically ill patients with sepsis has been associated with better target attainment and decreased hospital mortality [20, 21]. For example, Rhodes et al. showed that receipt of prolonged infusion of piperacillin/tazobactam (TZP) is associated with an improved antimicrobial exposure and reduced mortality across diverse cohorts of severely ill patients [22].

In addition to prolonged infusion, therapeutic drug monitoring (TDM) is increasingly used to guide dosage in order to maximise the probability of target attainment and to prevent under- and overdosing [23]. Recently, De Waele et al. [24] investigated the effect of TDM on 41 patients with TZP or meropenem therapy in a randomised controlled trial (RCT). The intervention group underwent daily TDM, with dose adjustment performed as necessary. The predefined PK/PD target was 100% f T_>4MIC_ (percentage of time during a dosing interval that the free [f] drug concentration exceeded 4 times the MIC). The study showed that only 21% of patients had sufficient serum piperacillin concentrations on the first day after initiation of therapy. In the TDM intervention group, dose adjustments had to be made in 76% of the patients. On the third day after the start of treatment, 58% of patients with TDM had reached the target concentration. In contrast, in the patient group without TDM, this was the case in only 16% of patients. Patients in the control group without TDM also had lower median piperacillin baseline concentrations than patients receiving TDM-guided therapy (26 vs. 40 mg/L). No difference in patient outcome was observed.

In summary, there is compelling evidence that there is altered antibiotic pharmacokinetics in critically ill patients resulting in an inappropriate antimicrobial exposure which negatively affects clinical outcomes. Prolonged infusion and TDM-guided therapy can improve antimicrobial exposure and hence clinical outcomes.

### Hypothesis

Our hypothesis is that continuous infusion of TZP guided by daily TDM in patients with severe sepsis or septic shock will result in a greater resolution of organ dysfunction and hence better clinical outcome compared to continuous infusion of TZP without TDM guidance.

## Methods/design

### Overview of trial design

TARGET is an investigator-initiated, multi-centre, parallel-group, single-blinded (trial participants) RCT. Participants started on TZP will be randomly allocated to receive the substance by continuous infusion guided by daily TDM of piperacillin (experimental intervention) or by continuous infusion of the daily dose without TDM (control group). Approval has been obtained from the leading Institutional Review Board at Jena University Hospital (ref. 4825-06/16), all relevant Institutional Review Boards of participating study sites and the Federal Institute for Drugs and Medical Devices (EudraCT: 2016-000136-17, ref. 4041358). A Standard Protocol Items: Recommendations for Interventional Trials (SPIRIT) checklist is provided as Additional file 1.

### Setting

**Fig. 1:**
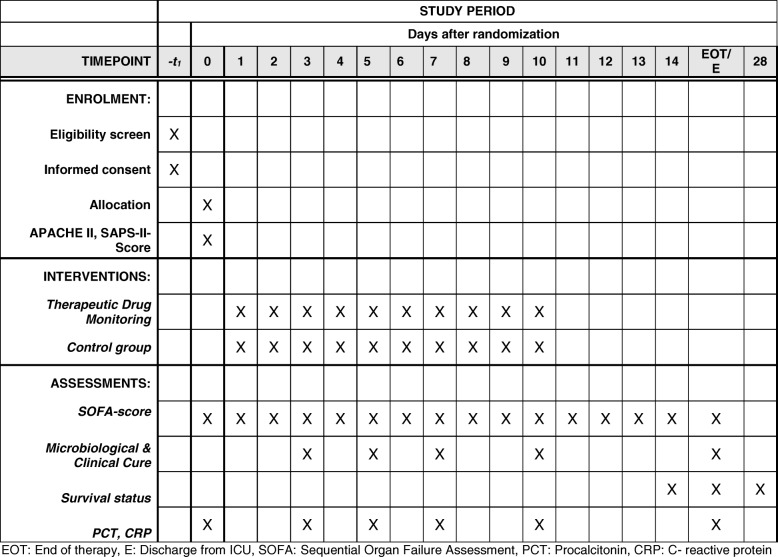
Schedule of enrolment, interventions and assessments

**Fig. 2:**
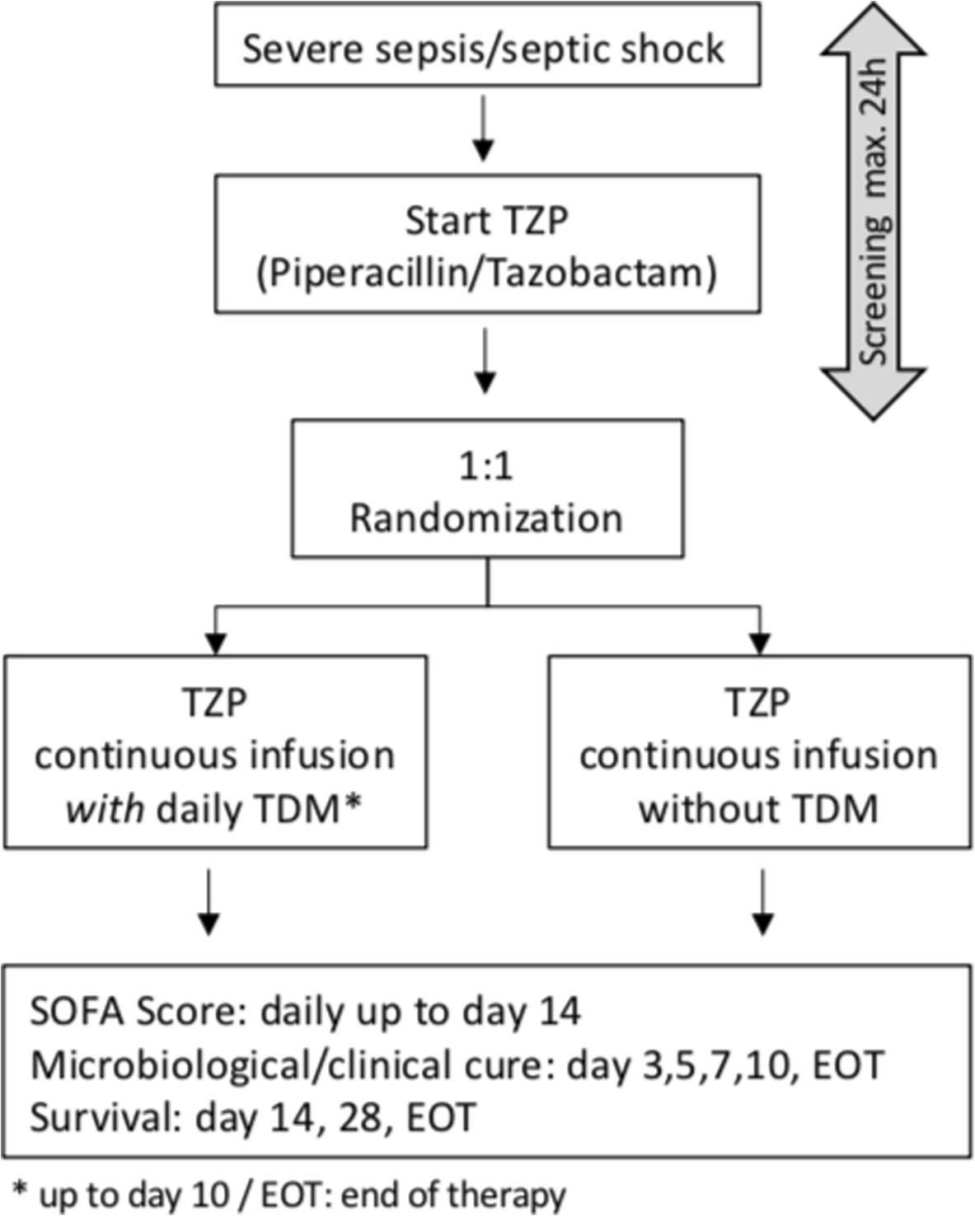
Flowchart

The trial will be conducted in several centres across Germany, university hospitals and academic teaching hospitals (see Table 1). The schedule of enrolment, interventions and assessments is shown in Figs. 1 and 2.

**Table 1:** Proposed participating sites

Department of Anesthesiology and Intensive Care Medicine, Charité-Universitätsmedizin Berlin	
Department of Intensive Care, University Medical Center Hamburg-Eppendorf, Hamburg	
Department of Anesthesiology, University Hospital Ulm, Ulm	
Department of Anesthesiology and Intensive Care Medicine, Jena University Hospital; Jena	
Department of Anesthesiology, Special Pain Management and Intensive Care Medicine, General Hospital of Heidenheim, Heidenheim	
Department of Anaesthesiology and Intensive Care Medicine, University Hospital of Cologne, Cologne	
Department III of Internal Medicine, University Hospital of Cologne, Cologne	
Department of Anesthesiology, Heidelberg University Hospital, Heidelberg	
Department of Anesthesiology, Intensive Care, Transfusion and Emergency Medicine and Pain Therapy, Bethel Hospital Bielefeld, Bielefeld	
Department of Anesthesiology and Intensive Care Medicine, Hospital of Sindelfingen, Sindelfingen	
Department of Internal Medicine I, Intensive Care Unit, University Hospital of Würzburg, Würzburg

### Primary objective

The primary objective is to determine whether continuous infusion of TZP guided by daily TDM in patients with severe sepsis or septic shock will have a benefit on organ function as measured by the Sequential Organ Failure Assessment (SOFA) score compared to continuous infusion of TZP without TDM guidance. The SOFA score has been recommended in the 2006 European Medicines Agency (EMA) publication “Guideline on clinical investigation of medicinal products for the treatment of sepsis” (CHMP/EWP/4713/03) for the assessment of the severity of disease in patients with sepsis [25]. The scale of the SOFA score ranges from 0 to 24, with higher scores indicating a greater severity of organ failure. Subscores of SOFA range from 0 to 4 for each of the 6 organ systems, with an aggregate score of 0 to 24. The mean SOFA score is calculated as the mean of all daily SOFA scores and can be used as a surrogate for the assessment of mortality [26]. In this study the primary efficacy endpoint is the mean total SOFA score measured from day 1 after randomisation until day 10 or discharge from the intensive care unit (ICU) or death, whichever comes first [27, 28].

### Secondary objectives

Secondary objectives aim at comparing the clinical and microbiological success of each treatment approach. Visits for clinical cure (CC) and microbiological cure (MC) will be performed on days 3, 5, 7, 10 and 14 after randomisation and at the end of therapy (EOT) with TZP and on day of discharge from the ICU, if discharge is before day 14 (Tables 2 and 3). In addition, the influence of each treatment approach on several other secondary outcomes will be assessed:

- SOFA subscores
- Survival at day 28 after randomisation
- Duration and cumulative dosage of antibiotic therapy
- Number of days free of antibiotic therapy
- Length of hospital stay
- Length of ICU stay
- Cost of antibiotic therapy
- Number of days free on ventilator, vasopressor or renal replacement therapy
- Emergence of antibiotic-resistant bacteria
- PK/PD indices
- Neurological outcome
- Assessment of safety

**Table 2:** Definition of clinical cure

Clinical cure	
Resolution	Disappearance of all signs and symptoms related to the infection AND No requirement for additional antibiotic treatment (except as part of de-escalation strategy) for the disease to be examined AND No initiation of antibiotic treatment for the disease to be investigated within 48 h after completion of the study drug
Improvement	Marked or moderate reduction in the severity and/or number of signs and symptoms of infection AND No requirement for additional antibacterial treatment (except as part of de-escalation strategy) for the disease to be examined AND No initiation of antibiotic treatment for the disease to be investigated within 48 h after completion of the study drug
Failure	Signs and symptoms of infection persist or increase in comparison to baseline, or additional antibiotic treatment becomes necessary for the disease to be investigated

**Table 3:** Definition of microbiological cure

Documented microbiologic eradication	Elimination of the putative pathogen from repeated cultures of the site of infection
Presumed microbiologic eradication	Disappearance of acute signs and symptoms related to the infection and no culture results available
Documented microbiologic persistence	Persistence of the original pathogen from the original site of infection
Presumed microbiologic persistence	Clinical failure and no culture results from the site of infection available
Relapse	After initial eradication, isolation of a pathogen from the original site of infection within 14 days of randomisation
Superinfection	Clinical failure or improvement and isolation of a pathogen not present at baseline
Colonisation	Acquisition of yeast or bacteria not associated with features of infection
Indeterminate	Any patients who could not be classified into one of the forementioned definitions

### Eligibility criteria

#### Participant inclusion criteriaThe inclusion criteria are as follows:

- Age 18 years or older
- Severe sepsis or septic shock (defined according to published criteria [29], however without the prerequisite of existing systemic inflammatory response syndrome (SIRS) criteria)
- Patients will be eligible for study enrolment if the onset of the syndrome is not more than 24 h prior to randomisation
- TZP used to treat the infectious episode
- Written informed consent of the patient or representative

#### Participant exclusion criteriaThe exclusion criteria are the following:

- Pregnancy/lactation
- Known hypersensitivity to beta-lactam antibiotics or to any of the other ingredients of the study medication
- Treatment with TZP > 24 h prior to randomisation
- Receiving palliative or supportive treatment only, at the time of assessment for eligibility
- Patient has an underlying process likely to result in death before 28 days of follow-up
- Impaired liver function (Child-Pugh C)
- Participation in a clinical trial (including participation in TARGET previously)
- Piperacillin measurement not possible within 24 h after randomisation

The additional exclusion criterion “Renal insufficiency (acute or chronic) and renal replacement therapy or expected renal replacement therapy within the following 6 h after randomisation” was deleted in December 2017 due to a low recruitment rate.

### Randomisation

The allocation ratio between the two arms of the study is 1:1. The randomisation list will be generated by a computer-based algorithm (nQuery Advisor 7.0; Statistical Solutions, Farmers Cross, Ireland) in advance. Thereafter, the list will be implemented in an Internet-based randomisation tool developed by the Jena University Hospital Center for Clinical Studies. The list will be stratified only by centre. Within each stratum (centre) we will use blocking to get similar numbers in each group per centre at any time during recruitment. To prevent predictability, the block size varies and will be kept confidential. Moreover, randomisation will be performed via the Internet-based randomisation tool to ensure allocation concealment.

### Interventions

Participants will be randomly allocated to receive TZP by continuous infusion guided by daily TDM of piperacillin (experimental therapy arm) or by continuous infusion of TZP without TDM (control group). The study drug will be administered via a syringe pump (4.5 g of TZP in 50 ml NaCl 0.9% or water for injections) with an infusion rate depending on the intended total 24-h dose of TZP.

#### Experimental therapy arm

Participants in the experimental therapy arm will receive TZP by continuous infusion guided by daily TDM of piperacillin. Continuous infusion of TZP will be started immediately after administration of a loading dose (4.5 g TZP) with an infusion speed of 6.3 ml/h in patients with an estimated glomerular filtration rate (eGFR) > 20 ml/min, corresponding to a total daily dose of 13.5 g TZP. In patients with an eGFR < 20 ml/min, a total daily dose of 9 g will be administered. Beginning on day 1 after randomisation, dosing of TZP will be guided by TDM of piperacillin. TDM will be performed once daily (at least from Monday to Friday) with result notification and dose adjustment if necessary on the same day. Due to the linear kinetics of the test substance, the dose is adjusted by means of a ratio equation. However, dose adjustment will always be done taking into account other clinical parameters, e.g. initiation of renal replacement therapy or recovering of renal function. The duration of the intervention depends on the duration of TZP therapy, which is at the discretion of the treating physician; however, it will not be later than day 10 after randomisation. To maximize the likelihood of clinical cure and the chance to achieve sufficient study drug concentrations at the site of infection, the primary target for this study is 100% f T_>4MIC_. Susceptibility data for isolated pathogens will be used to establish the MIC target. Antimicrobial susceptibility tests will be performed in the clinical microbiology laboratory of each participating hospital, according to local antibiotic susceptibility testing methods. Until MIC data are available and for patients where no pathogen can be isolated, the MIC breakpoint for *Pseudomonas aeruginosa* (16 mg/L) will be chosen in consideration of a “worst-case scenario”. See Table 4 for details of dosing instructions in the experimental therapy arm.

**Table 4:** Piperacillin/tazobactam (TZP) dosing instructions for experimental therapy arm

Loading dose (LD)	Patients without TZP therapy within 24 h prior to randomisation: 4.5 g TZP in 30 min	
	Patients with TZP therapy within 24 h prior to randomisation: at the discretion of the physician (depending on when last dose of TZP was administered)	
Continuous infusion (onset after finishing LD to first dose adjustment)	eGFR ≥20 ml/min 13.5 g/24 h	
	eGFR < 20 ml/min 9 g/24 h	
Dose adjustment - Start: day 1 after randomisation (optional on day of randomisation)	MIC	Piperacillin target concentration^a^ (mg/L)
	Unknown pathogen *or* pathogen with MIC ≤16 mg/L	80 [64–96]
	Pathogen with MIC ≤8 mg/L	40 [32–48]
	Pathogen with MIC ≤4 mg/L	20 [16–24]

Piperacillin population pharmacokinetics: *t*1/2 = 1 h, Vd = 18 L, Cl = 12.5 L/h, free fraction = 0.81

^a^ MIC/0.81 * 4 = target concentration [+/− 20%]

#### Standard care arm

Participants in the standard care arm will also receive TZP by continuous infusion. Continuous infusion of TZP will be started immediately after administration of a loading dose (4.5 g TZP) with an infusion speed of 6.3 ml/h in patients with an eGFR > 20 ml/min, corresponding to a total daily dose of 13.5 g TZP. In patients with an eGFR < 20 ml/min, a total daily dose of 9 g will be administered. Daily dose adjustment will be performed according to current renal function as measured with the Cockroft-Gault formula or type of renal replacement therapy. In patients with renal impairment or renal replacement therapy, the total daily dose of TZP will be adjusted according to the recommendations of the Summary of Product Characteristics. See Table 5 for details of dosing instructions in the standard therapy arm. Blood samples for TDM will be obtained daily, starting on day 1 after randomisation, but treating physicians are blinded to the results. The duration of the intervention depends on the duration of TZP therapy, which is at the discretion of the treating physician; however, it will not be later than day 10 after randomisation. In addition, high qualitative samples (i.e. storage at − 80 °C) will be collected and stored for future analyses under standardised quality controlled conditions in the Integrated Biobank Jena in both groups.

**Table 5:** Piperacillin/tazobactam (TZP) dosing instructions for standard care arm

Loading dose (LD)	Patients without TZP therapy within 24 h prior to randomisation: 4.5 g TZP in 30 min
	Patients with TZP therapy within 24 h prior to randomisation: at the discretion of the physician (depending on when last dose of TZP was administered)
Dose adjustment	According to current renal function (GFR) as measured with Cockroft-Gault formula or type of renal replacement therapy: • eGFR ≥20 ml/min or CRRT or SLED: 13.5 g/24 h • eGFR < 20 ml/min or IHD: 9 g/24 h

*IHD* intermittent haemodialysis, *CRRT* continuous renal replacement therapy, *SLED* sustained low-efficiency dialysis

### Sample analysis

Measurement of piperacillin concentration will be performed on site in study centres with either validated high-performance liquid chromatography (HPLC) or validated liquid chromatography with tandem mass spectrometry (LC-MS/MS). Prior to study initiation interlaboratory tests will be performed. Basically, the tests are carried out following the recommendations of the laboratory guidelines of the EMA [30] and the US Food and Drug Administration (FDA) [31], which provide a mean deviation in the rule of method validation of +/− 15%.

### Data entry and storage

Data collection will be conducted by trained staff at each study site, and data will be entered into a web-based clinical trial database system (OpenClinica, LLC, Waltham, MA USA). Information to be collected via the case report form includes demographic data, patient characteristics, trial characteristics, co-morbidities and risk factors, infection parameters, antibiotic data, clinical observations and microbiological data and outcome data. The database will contain validation ranges to minimise the chance of data entry errors. An audit trail will maintain a record of the following: initial entries and changes made, reasons for change, time and date of entry, user name of person who made the change. Data queries will be raised by the data manager, study monitor and project manager, and missing data or suspected errors will be raised as data queries and resolved prior to database lock and analysis. The database will contain in-line capability so that these queries and answers are logged as part of the audit trail. Personnel trained by the Jena University Hospital Center for Clinical Studies will conduct monitoring for the study. The Jena University Hospital Center for Clinical Studies will develop and manage the trial database and conduct the data analyses. Only study personnel (e.g. principal investigator, data manager, statisticians) from Jena University Hospital will have access to the final trial dataset.

### Safety monitoring plan

A Data Safety Monitoring Board (DSMB) will be established, comprising two independent infectious disease physicians and one independent statistician with statistical support and relevant data listings provided to them by the Jena University Hospital Center for Clinical Studies. The DSMB receives information about the trial progress, amendments and listings of safety-relevant items including serious adverse events (SAEs) and suspected unexpected serious adverse reactions (SUSARs) on a yearly basis. After examining the available data, the DSMB makes recommendations regarding continuation, modification or discontinuation of the clinical trial.

### Antimicrobial therapy

The use of combination therapy with additional antimicrobials is allowed in the study. The decision to carry out is incumbent on the attending physician. An escalation or de-escalation of antimicrobial therapy will be allowed at any time during the study.

### Protocol deviations

All important protocol deviations occurring after randomisation will be listed in the Clinical Study Report, tabulated by subject and recruitment site. The final assignment of participants to the per-protocol analysis population will be made at a blinded protocol violation review meeting prior to database lock.

### Quality assurance and safety

The information entered into the electronic case report form (eCRF) at the trial site is regularly systematically checked for completeness, consistency and plausibility by routines implemented in data capture software and by centralised monitoring. Agreement of study data with source data and compliance with the informed consent process are verified by external monitors (Center for Clinical Studies). Safety of the study medication is assessed by reporting of adverse events, SAEs and SUSARs. According to German regulations, safety reports are forwarded to the authorities and ethics boards. A DSMB will receive a descriptive analysis regularly to assess the safety of the study intervention.

### Proposed sample size/power calculations

The sample size is calculated for the individually averaged SOFA score over time as described for the primary endpoint. According to data from the SepNet study group (VISEP [32] and MAXSEP [33] RCTs, *n* = 1137 patients), a 1.4-point-lower SOFA score in the intervention group compared to the control group would be of clinical relevance. Assuming a standard deviation of 3.8 points (SepNet data), the difference can be transferred to an effect size of 0.368. To demonstrate this effect with 80% power using a two-sample *t* test at a 5% two-tailed significance level, 117 patients per study arm are required (using the software nQuery Advisor 7.0). From the experiences of the SepNet studies, a dropout rate of 15% is expected. In order to achieve the necessary number of cases for the analysis, 276 (2 × 138) patients must be randomised.

### Statistical analysis

Data will be reported according to the Consolidated Standards of Reporting Trials (CONSORT) guidelines for reporting of randomised trials. Group-specific baseline data and endpoints will be described by appropriate statistical measures (mean, standard deviation, 25th, 50th, 75th percentile, interquartile range, absolute and relative frequencies).

#### Primary analysis

The primary endpoint is the mean total SOFA score. It enters the analysis as an individual average over the course of day 1 after randomisation until day 10 or discharge from the ICU or death, whichever comes first. It will be analysed in the intent-to-treat population by a linear mixed model with intervention, total SOFA score and renal failure at baseline as fixed factors and centre as random factor. The group difference with 95% confidence interval will be estimated to quantify the effect of the intervention. In case of relevant numbers of missing values, imputation techniques will be applied in a sensitivity analysis. A formal interim analysis with statistically motivated stopping rules is not intended.

#### Secondary endpoints

Secondary endpoints will be analysed in an exploratory manner. Continuous data will be tested by appropriate methods depending on the scale of the endpoint, i.e. linear models or non-parametric methods. Categorical secondary endpoints will be tested by the chi-square test or Fisher’s exact test. For time-to-event endpoints, survival analysis will be performed. Longitudinal binary and ordinal endpoint data will be analysed by generalised linear mixed models. Adverse events will be reported for each group by absolute and relative frequencies.

#### Subgroup analysis

An exploratory subgroup analysis for the primary and selected secondary endpoints will be based on modelling the interaction with the intervention. Subgroups of interest are patients with/without infection with pathogens displaying a high MIC (not yet specified), patients with/without augemented renal clearance (as defined by a measured creatinine clearance > 130 ml/min/1.73 m^2^) and patients grouped by infectious foci (pulmonary, bloodstream infection, intra-abdominal, bone/soft tissue). Moreover, selected endpoints will be analysed in the population of patients who survived at least 48 h.

### Stopping rules

The entire trial can be terminated prematurely by the sponsor at any time for medical and ethical reasons (i.e. recommendation by the DSMB). The sponsor may terminate participation of a study site if inadequate protocol adherence is repeatedly observed, the quality of the data is deficient or the recruitment is insufficient. The study can be terminated for individual patients if the patient or the legal representative withdraws informed consent, severe side effects of the study medication are observed or the treating physician assesses the trial participation as being detrimental for the patient.

### Ethical considerations

All trial participants will conduct the study in accordance with local laws and International Conference on Harmonisation (ICH) guidelines for Good Clinical Practice (GCP). The trial was approved by the ethics committee of each participating institution and by Germany’s Federal Institute for Drugs and Medical Devices. Written informed consent will be obtained from all patients or their legal representative by study physicians. For patients in whom prior consent cannot be obtained because of critical illness or the use of sedative or anesthetic drugs and to enable early antibiotic therapy, the ethics committees approved a provision for delayed consent. In such cases, a surrogate decision-maker will be fully informed as soon as possible. For participants enrolled under this provision, consent to continue with study participation will be obtained from the subject or person responsible as soon as practicable after study enrolment or the patient will be removed from the study and all study procedures will be terminated. The trial design takes several patient safety considerations into account. The decision on prescription of the trial drug is at the discretion of the treating physician. Patients receive an effective and safe antibiotic treatment, recommended by the current sepsis guidelines. In the control group dosing of the study drug will be carried out according to manufacturers’ recommendations. To maximize the likelihood of clinical cure, we define the primary TDM target in the intervention group as 100% f T_> 4MIC_. Whether this target is too “aggressive” is unclear, but it constitutes the option with the maximum likelihood of clinical cure hitherto proposed and is unlikely to result in toxicity. Until MIC data are available and for patients where no pathogen can be isolated, the MIC breakpoint for *Pseudomonas aeruginosa* (16 mg/L) will be chosen in consideration of a “worst-case scenario”. With these safety precautions a maximum of safety is guaranteed for the study participants. In addition, stability issues of the study drug and possible incompatibility with other substances will be addressed. All study participants are insured according to the requirements of the Medicinal Products Act.

## Discussion

Inappropriate antimicrobial exposure has been identified to negatively affect clinical outcomes in patients with sepsis. One strategy to overcome the problem of under- and overdosing and hence improve antimicrobial exposure is therapeutic drug monitoring (TDM) guided therapy. This study will address for the first time whether TDM-guided therapy will improve clinical outcome in patients with severe sepsis or septic shock treated with TZP administered by continuous infusion. The primary endpoint will be resolution of organ dysfunction, measured by the mean total SOFA score. Several studies could show that the mean total SOFA score can be used as a surrogate for the assessment of mortality.

In the study of Ferreira et al. [27] for example, patients with a mean SOFA score of 4.1–5 had a significantly higher ICU mortality than patients with a score of 3.1–4 (73% vs. 36%). As shown in previous studies, a 1.4-point-lower mean SOFA score in the intervention group compared to the control group is therefore considered to be of clinical relevance.

Besides the effect of TDM-guided therapy on clinical outcomes, the study will address several other important questions, including the probability of target attainment in patients with continuous infusion of TZP without TDM. One concern of continuous infusion of antibiotics without TDM is the risk of underdosing throughout the entire dosing interval, especially in patients with augemented renal clearance and/or underlying pathogens with a high MIC. In addition to underdosing, the correlation between antimicrobial exposure and the risk for TZP-associated toxicity (e.g. delirium) and a cost-effectiveness analysis of TDM will be assessed.

### Trial status

The TARGET trial randomised its first patient on 26 January 2017. The aim is for recruitment for the study to be completed by late 2019.

## Additional file

Additional file 1: SPIRIT 2013 checklist: recommended items to address in a clinical trial protocol and related documents. (DOC 121 kb)

## Data Availability

Not applicable.

## Abbreviations

CC: Clinical cure
DSMB: Data Safety Monitoring Board
eCRF: Electronic case report form
EMA: European Medicines Agency
FDA: Food and Drug Administration
GCP: Good Clinical Practice
MC: Microbiological cure
MIC: Minimum inhibitory concentration
PD: Pharmacodynamic
PK: Pharmacokinetic
RCT: Randomised controlled trial
SAE: Serious adverse event
SOFA: Sequential Organ Failure Assessment
SUSAR: Suspected unexpected serious adverse reaction
TDM: Therapeutic drug monitoring
TZP: Piperacillin/tazobactam
